# Continuous Monitoring of Suspended Particulate Matter in Tropical Inland Waters by High-Frequency, Above-Water Radiometry

**DOI:** 10.3390/s22228731

**Published:** 2022-11-11

**Authors:** Henrique Dantas Borges, Jean-Michel Martinez, Tristan Harmel, Rejane Ennes Cicerelli, Diogo Olivetti, Henrique Llacer Roig

**Affiliations:** 1 Institute of Geosciences, Campus Darcy Ribeiro, University of Brasília, ICC-Ala Central, Brasília CEP 70910-900, Brazil; 2 Institut de Recherche pour le Développement (IRD), Géosciences Environnement Toulouse (GET), UMR5563, Centre National de la Recherche Scientifique (CNRS), Université Toulouse 3, 14 Avenue Edouard Belin, 31400 Toulouse, France

**Keywords:** suspended sediment concentration, hyperspectral, remote sensing, water color, reflectance

## Abstract

Water and sediment discharges can change rapidly, and low-frequency measurement devices might not be sufficient to elucidate existing dynamics. As such, above-water radiometry might enhance monitoring of suspended particulate matter (SPM) dynamics in inland waters. However, it has been barely applied for continuous monitoring, especially under partially cloudy sky conditions. In this study, an in situ, high-frequency (30 s timestep), above-water radiometric dataset, collected over 18 days in a tropical reservoir, is analyzed for the purpose of continuous monitoring of SPM concentration. Different modalities to retrieve reflectance spectra, as well as SPM inversion algorithms, were applied and evaluated. We propose a sequence of processing that achieved an average unsigned percent difference (UPD) of 10.4% during cloudy conditions and 4.6% during clear-sky conditions for Rrs (665 nm), compared to the respective UPD values of 88.23% and 13.17% when using a simple calculation approach. SPM retrieval methods were also evaluated and, depending on the methods used, we show that the coefficient of variation (CV) of the SPM concentration varied from 69.5% down to 2.7% when using a semi-analytical approach. As such, the proposed processing approach is effective at reducing unwanted variability in the resulting SPM concentration assessed from above-water radiometry, and our work paves the way towards the use of this noninvasive technique for high-frequency monitoring of SPM concentrations in streams and lakes.

## 1. Introduction

Aquatic environments, especially rivers, are notoriously dynamic, and water constituents can vary within a very short time span. Suspended sediment transported in rivers and other water bodies can vary considerably over different time scales. According to Vercruysse et al. [1], this variation is due to interactions between: hydro-meteorological events, the sediment source, terrain disturbances, and human action. High-flow events are responsible for a large portion of a river’s total sediment load [2], and it has been estimated that 40–80% of the total river sediment load is transported in 2% of the time [3]. As such, a measurement device that could present the low-frequency acquisition rate would underestimate the suspended sediment fluxes, as well as the fluxes of associated elements that are transported on the particles such as nutrients, heavy metals, and pathogens.

Having a complete picture of the sediment load variability can be extremely important for reservoir sediment management, particularly in dams located in rivers with high sediment concentrations, as siltation is one of the most significant operational challenges in the usage of hydropower [4]. This is a problem worldwide: for instance, in Asia, a reduction of 80% of the operational storage volume is expected by 2035, and in Europe by 2080 [5,6].

Water bodies contain spectral information that can be converted into data about its optically active constituents (OACs), such as phytoplankton, colored dissolved organic matter (CDOM), and suspended particulate matter (SPM). For this reason, remote-sensing instruments have been successfully used as a tool to monitor the concentration of OACs in such water bodies [7,8,9,10]. Their application in the estimation of suspended solids concentration is already well established in the literature, both with field radiometric sensors and with the use of orbital sensors [11,12,13,14]. However, even though satellite images are advantageous from the point of view of their spatial coverage, they have limitations regarding their temporal and spectral resolution. Most available satellite sensors can offer, at best, an image every day from the same location and are limited by the occurrence of cloud coverage that can severely reduce the availability of spaceborne sensor imagery during rainy episodes and high-flow events. The collection of airborne radiometric data enables higher acquisition frequency, but it still requires significant human resources for operation and cannot be used as a permanent monitoring alternative [15,16]. Conventional water quality monitoring is based on the collection of frequent water samples for analysis using local operators or automatic sampling instruments which require frequent maintenance, being impossible to deploy at a large scale within the catchments. Consequently, the estimation of the suspended sediment load being transported by a given river can suffer from very significant inaccuracies when using sampling methods or satellite data [17,18].

For this reason, continuous turbidity measurements through underwater probes are generally the most utilized method for the estimation of river SPM as, once calibrated, they do not require water sampling [19,20,21]. Hence, to retrieve SPM from a turbidity record, a concentration curve that relates the turbidity measurements to SPM must be created using samples collected during a period long enough to cover all kinds of hydrological conditions. The absence of this kind of calibration procedure can create errors in the final estimated SPM values, due to the turbidity measurement sensitivity to sediment grain size variability and to the relative organic fraction. Furthermore, turbidity probes require direct contact with the water, leading to rapid degradation (i.e., biofouling) of the measurements without appropriate and frequent maintenance [22].

Given this context, the utilization of field spectroradiometric stations for measuring SPM appears as an innovative alternative to acquire high-frequency data without any invasive sampling. Although some field spectroradiometric stations have been used for the continuous monitoring of water reflectance [23,24,25], they have been mostly used for the purpose of satellite sensor calibration and validation [26,27], not for the continuous measurement of water constituents in relation to watershed monitoring. Additionally, existing measurement protocols [28,29,30] generally limit this measurement scheme to optimal environmental illumination conditions, such as optimal sun elevation and relative azimuth angles, as well as clear-sky conditions. Data degradation in suboptimal conditions is mostly caused by uncorrected features originating from sky and sun reflection on the water surface (i.e., “glint effect”). For this reason, different approaches have been proposed to correct such errors, such as: (1) calculating the most appropriate value of the surface reflection factor (𝜌) [28,31]; (2) using the dark pixel assumption at certain wavelengths to infer the effect of glint [32]; (3) exploiting some spectral features of the water reflectance, which is nearly invariant in the near-infrared [33]; and (4) the use of pure-water absorption features in the reflectance spectra to infer the amount of glint to be corrected [34].

There is a need to further assess the capacities of field continuous radiometry for inland water quality monitoring, exploring the variability of the remote-sensing reflectance (R_rs_) in a broad range of weather and illumination conditions that include large irradiance variability and contrasted weather conditions corresponding to nearly the whole daytime. For this objective, we developed an experiment that consisted of acquiring continuous, hyperspectral radiometric data from sunrise to sunset in a reservoir located in the tropics (central Brazil) at the end of the rainy season with very different conditions corresponding to fully cloudy conditions, tropical rain events, or to bright days with small solar zenith angle (θ) conditions at midday.

This article presents a methodology to retrieve continuous SPM records from hyperspectral data based on the detection of broad weather conditions, the benchmarking of physically based reflectance correction schemes for sun/sky glint, followed by statistical noise reduction procedures in order to produce robust R_rs_ time series acquired at a 30 s time scale. The R_rs_ data are then processed for SPM calculation and an assessment of the method error is presented. Instead of looking at a water body presenting varying water quality, we took the reverse option of looking at a stable water body corresponding to the dam area of a large reservoir. Large reservoirs present very stable conditions in their downstream parts as strong sedimentation occurs upstream, and in such reservoirs, turbidity may vary by only a few percent during an entire year [35]. The stability of the water SPM load across the whole experiment made it possible to identify and study all the artifacts inherent to R_rs_ acquisition and processing and to quantify their relative importance.

In this work, a measurement and processing scheme is proposed, in which different complementary correction methods are applied to the same set of radiometric data that are capable of measuring water SPM under varying illumination conditions. Particularly, to do this we will: (1) collect controlled high-frequency continuous radiometric measurements from a reservoir; (2) test different methodologies for obtaining R_rs_; (3) process the estimated R_rs_ to reduce noise and unwanted variability; and (4) retrieve the high-frequency, continuous concentration of SPM. As such, we aim to evaluate these correction methods to identify the best performing ones and to propose a processing pipeline that can obtain continuous, high-frequency Rrs spectra and SPM concentrations in suboptimal observation conditions.

## 2. Materials and Methods

### 2.1. Test Site

**Figure 1:**
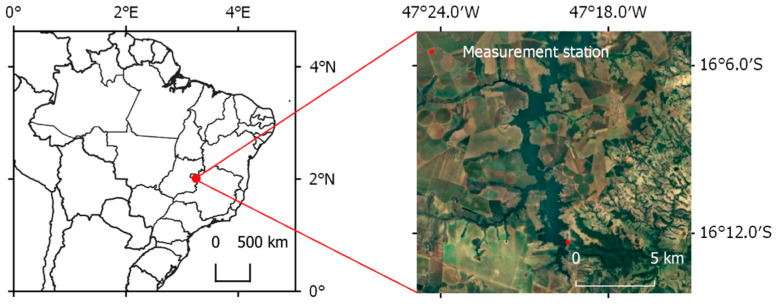
Map showing measurement station location in central Brazil.

The measurements were taken in the Queimado Reservoir located at the outlet of the Preto River catchment, which is a tributary of the São Francisco River in Brazil, as shown in Figure 1. The reservoir is used for both hydropower generation and irrigation and is maintained by the CEB and CEMIG companies. The Preto River catchment is an area of 3600 square kilometers, where the mean annual rainfall is 1336 mm and the rainy season extends from November to April. The Queimado Reservoir itself has an area of 36 square kilometers.

We gathered hourly data collected by an automated meteorological station from INMET (Instituto Nacional de Meteorologia), located 33 km from the measurement station. The average temperature during the radiometric experiment was 22.4 °C (max 29.8 °C; min 17.7 °C) and average wind speed was 1.9 m/s, with a std of 1.1 m/s. Although gusts of wind of up to 10.3 m/s were recorded, the upper quartile wind speed was 2.4 m/s. Precipitation was recorded for 7 of the 18 days of measurement, totaling 24.8 mm of rain.

While radiometric measurements were being acquired, the station was visited on three different days, in which water samples were collected at approximately midday to determine the SPM concentration at the dam surface at the experiment location. The concentration of total solids in suspension was then determined by the method described by the APHA [36]. The samples were filtered through cellulose acetate filters, previously dried for 24 h at 60 °C. After filtering, the filters were dried again for 24 h at 60 °C. The suspended solids concentration was then determined by the difference in weights before and after filtration. All three samples found a SPM concentration of 1.0 g/m^3^.

### 2.2. Radiometric Measurements

Radiometric measurements were performed continuously for 18 days (12 April 2019 to 30 April 2019) using TriOs RAMSES radiometers operating in the 350–950 nm spectral range. One radiometer was mounted with a cosine collector for irradiance measurements, and two other radiometers were equipped for radiance measurements with a field of view of 7°. All the radiometers were synchronized to simultaneously record a measurement every 30 s. Measurements were collected from an intake tower located in the reservoir near the dam, with a water column of about 10 m eliminating any possible influence from the reservoir bottom or shore. The radiometers were located about 7 m above the water surface.

We utilized a viewing angle of 40° (angle between the radiometer and a downward vertical axis) for the radiance radiometers, whereas the irradiance radiometer was pointed towards the zenith following the protocol proposed by Mobley [28]. The radiometer’s azimuth remained fixed during the whole experiment at 240° (north clockwise), meaning that the radiometer-sun relative azimuth was constantly varying as a function of the apparent sun movement in the sky. This relative azimuth was chosen to both reduce shading from the structure to which the station was mounted and to maximize the time in which the relative azimuth was larger than 90°. We chose not to use rotating platforms as this kind of installation would be hardly feasible for water quality measurements in isolated conditions.

### 2.3. Data Processing

**Figure 2:**
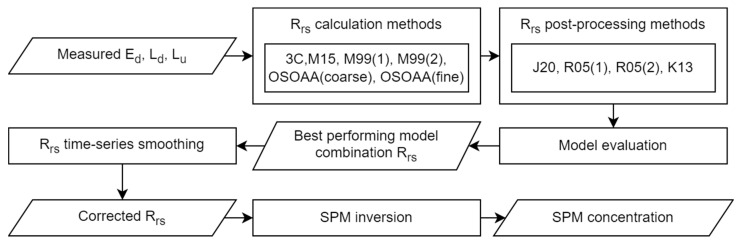
Data processing methodology schematic.

A specific evaluation scheme was designed to evaluate the respective performances of the different retrieval modalities to be applied to the raw radiometric data for the determination of R_rs_. This scheme was also completed using two different methodological approaches to obtain the SPM concentration from R_rs_. These methods are described below and are shown schematically in Figure 2.

The processing scheme for obtaining R_rs_ consists of three main steps: (1) direct retrieval of R_rs_ from the radiometric data (E_d_, L_d_, and L_u_); (2) further postprocessing to correct for residual glint effects in R_rs_; (3) time-series smoothing based on a smoothing filter applied to the acquired time-series. The methods used in each step are described in the following sections.

#### 2.3.1. R_rs_ Calculation Methods

As proposed by Mobley (1999) [28], R_rs_ can be calculated as shown in Equation (1).

(1) $$R_{\mathrm{rs}}\left(\lambda \right)=\frac{L_{u}\left(\lambda \right)-{\rho *L}_{d}\left(\lambda \right)}{E_{d}\left(\lambda \right)}\left[{\mathrm{sr}}^{-1}\right]$$

where E_d_(λ) is the downwelling irradiance above the water surface, L_u_(λ) is the upwelling radiance above the surface water, and L_d_(λ) is the sky radiance, which is used to correct for the skylight reflection effect at the air–water interface. The above-water upwelling radiance L_u_ is the sum of the water-leaving radiance L_w_(0^+^) and of the surface radiance, L_surf_, originating from the sun and skylight (or cloud) reflected onto the air–water interface. Because only L_u_ is directly measurable, L_surf_ is, in its simplest formulation, assessed as L_surf_ = *ρ* L_d_, where *ρ* is a proportionality factor frequently referred to as the Fresnel surface reflectance factor. The factor ρ is not an inherent optical property of the surface and is dependent on sky conditions, wind speed, solar zenith angle, and viewing geometry, and varies with wavelength. The next four models described use different approaches to derive the value of *ρ*:

1. A value of *ρ* equal to 0.028 was assessed from the optical modeling for ideal conditions (i.e., perfectly plane surface) and for a viewing angle of 40° and relative azimuth of 135°. This method is henceforth referred to as M99(1) and stands as the simplest correction approach as it does not vary as a function of viewing geometry nor wavelength.
2. R_rs_ calculated using the *ρ* table from Mobley [28] and applied to Equation (1). The table offers specific *ρ*-values for combinations of wind speed, relative azimuth, and viewing angle. As such, to find *ρ*, relative azimuth values were determined at each measurement step, the viewing angle was 40°, and the wind speed was assumed to be 2 m/s for all data points. It should be noted that even though some meteorological wind data were available, it was not accurate enough to be used for shorter time scales; thus, an overall wind velocity average was preferred. Henceforth, it is referred to as M99(2).
3. R_rs_ calculated similarly to M99(2), but using the updated rho table published in [31]. Henceforth, it is referred to as M15.
4. Following the abovementioned approach, the *ρ*-factor was also computed using the radiative transfer code OSOAA [37]. Those computations enable us to directly handle the impact of the light polarization at play in the skylight reflection on the rough water surface [38]. Spectral *ρ*-factor values were computed for two aerosol-load cases: (i) a fine-mode aerosol model with a modal radius of 0.06 µm and (ii) a coarse aerosol mode with a modal radius of 0.6 µm. For both cases, simulations were performed for a series of aerosol optical thicknesses (from 0 to 1 at 550 nm), several wind speeds (0 to 12 m/s), and for a great number of viewing geometries corresponding to the sun zenith from 0 to 88° (increment 4°) and azimuth angles from 0 to 360° (increment 5°). Note that only clear-sky conditions were considered in those computations. In the rest of the article, the methods using the fine- or the coarse-mode aerosol are referred to as OSOAA(fine) and OSOAA(coarse), respectively.
5. The three-component method (hereafter referred to as 3C) exploits an approach in which the spectral dependence of the glint contribution is obtained by distinguishing three irradiance components: the direct solar irradiance, the diffuse molecular-scattered irradiance, and the diffuse aerosol-scattered irradiance. The 3C method combines an aquatic component, in which a semi-analytical, bio-optical model is used to estimate R_rs_ based on certain optical properties as well as boundary conditions and an atmospheric correction model. An optimization procedure is then used to minimize an objective function related to the differences observed between the modeled and measured values of L_w_/E_d_, which returns the values of the nine free parameters used in the aquatic and atmospheric models. R_rs_ is then determined by utilizing the four atmospheric free parameters to calculate a spectrally dependent glint offset and then find R_rs_ based on measured values of E_d_, L_u_, and L_d_. A more complete description of the model can be seen in the original work [39] as well as in the follow-up paper [40]. It should be noted that, although the 3C method is an R_rs_ calculation method in the sense that it takes radiometric data as input and outputs R_rs_ curves, it is also a postprocessing algorithm in the sense used in this article, as it was developed with the intent to correct spectra obtained in suboptimal conditions. For this reason, further postprocessing steps (see next section) used in the present study were not applied to the 3C model.

#### 2.3.2. R_rs_ Postprocessing Methods

1. The similarity spectrum, as described in Ruddick et al. [33]. In this method, we assume that the true R_rs_ is related to the measured R_rs_ by a flat error factor ε, as shown in Equation (2).
  (2) $${\;R\;}_{\mathrm{rs}}^{\prime }\left(\lambda \right)=R_{\mathrm{rs}}\left(\lambda \right)-\varepsilon$$
  This error factor ε can be estimated as:
  (3) $$\varepsilon =\frac{\alpha \cdot R_{\mathrm{rs}}\left(\lambda_{1}\right)-R_{\mathrm{rs}}\left(\lambda_{2}\right)}{\alpha -1}$$
  in which $\lambda_{1}$ and $\lambda_{2}$ are two suitably chosen NIR wavelenghts, and α is a related tabulated value provided by the authors. The authors suggest using two suitable pairs of wavelengths, (λ_1_, λ_2_) = (720 nm, 780 nm) and (780 nm, 870 nm), which are calculated with α = 2.35 and α = 1.91, respectively. Both pairs of wavelengths were tested in this study and are further referred to as R05(1) and R05(2).

2. The correction method proposed by [34]—further referred to as J20, which utilizes the relative height of the water absorption dip-induced reflectance peak at 810 nm—uses RHW as a baseline index. RHW can be calculated using Equations (4) and (5).
  (4) $$\;\mathrm{RHW}=R_{\mathrm{rs}}\left(810\right)-R_{\mathrm{rs}}^{\prime }\left(810\right)$$
  (5) $$R_{\mathrm{rs}}^{\prime }\left(810\right)={\;R}_{\mathrm{rs}}\left(780\right)+(R_{\mathrm{rs}}\left(840\right)-R_{\mathrm{rs}}\left(780\right))\times \left(810-780\right)/\left(840-780\right).$$

The authors then proposed a method to estimate R_rs_ at 810 nm, in which R_rs_(810) is empirically derived from RHW based on a synthetic dataset. As such, R_rs_(810) can be calculated with the following equation.

(6) $${\mathrm{estimatedR}}_{\mathrm{rs}}\left(810\right)=16,865.541\mathrm{RHW}³-52.728\mathrm{RHW}²+3.361\mathrm{RHW}\;$$

The value of Δ can then be calculated using Equation (7) and used to derive the corrected R_rs_ with Equation (8):

(7) $$∆={\;\mathrm{estimatedR}}_{\mathrm{rs}}\left(810\right)-R_{\mathrm{rs}}\left(810\right)$$

(8) $$J20R_{\mathrm{rs}}\left(\lambda \right)=R_{\mathrm{rs}}\left(\lambda \right)-∆$$

3. An adaptation of the method proposed by [32], in which we fit a power function of the R_rs_ values between the spectral ranges of 350–380 nm and 890–900 nm, and then subtract the values of the obtained power function from the original R_rs_. In the original work, the authors perform the correction directly on the reflectance values (L_u_/E_d_). Here, we apply the correction scheme to previously calculated R_rs_, as presented in the previous section. Henceforth, it is referred to as K13.

#### 2.3.3. R_rs_ Time-Series Smoothing

Following the first two processing steps, a moving median smoothing filter was then applied to the R_rs_ data. For a window n and timestep t, it was calculated using Equation (9). For this study, we chose n = 30 (15 min window).

(9) $$R_{\mathrm{rsmedian}}\left(\lambda ,t\right)=\mathrm{Median}\left(R_{\mathrm{rs}}\left(\lambda ,t-n\right),R_{\mathrm{rs}}\left(\lambda ,t-n+1\right),\ldots ,R_{\mathrm{rs}}\left(\lambda ,n-1\right),R_{\mathrm{rs}}\left(\lambda ,n\right)\right)$$

### 2.4. Validation

The assessment of each method was carried out via a statistical comparison of the unsigned percent difference (UPD) values, as shown in Equations (10) and (11).

(10) $$\mathrm{UPDday}=\frac{100}{N}\overset{N}{\underset{i=1}{\sum }}\frac{{\mathrm{Rrs}}_{i}-{\mathrm{Rrs}}_{\mathrm{ref}}\left(\lambda \right)}{{\mathrm{Rrs}}_{i}}$$

(11) $$\mathrm{UPD}=\frac{\mathrm{UPDday}}{\mathrm{Number}\;\mathrm{of}\;\mathrm{days}}\;$$

in which the reference remote-sensing reflectance (${\mathrm{Rrs}}_{reference}$) was determined, for each day, as the median of the R_rs_ collected with ideal illumination conditions during that day, and the i value refers to each individual R_rs_ spectra measured that day. The UPD is then calculated as the average of the UPD values obtained for each day.

The chosen validation procedure is based on the assumption that the magnitude of intraday variation in R_rs_ is almost totally due to the variation in observational parameters, such as weather and sun position, and not due to changes in water composition.

### 2.5. SPM Assessment

The processed R_rs_ was then used to measure SPM concentration using a simple band ratio formula. To do this, we chose two state-of-the-art methods which employ different approaches to estimate the SPM from the R_rs_ data. The first method, proposed by Nechad et al. [41], utilizes a semi-empirical formulation, in which the SPM concentration is obtained with Equation (12):

(12) $$\mathrm{SPM}=\;A\frac{R_{\mathrm{rs}}}{1-\frac{R_{\mathrm{rs}}}{C}}+B$$

in which A, B, and C are wavelength-dependent parameters that were empirically calibrated in the original study. Here, we utilize λ = 665 nm, for which the parameters are 355.85 (g∙m^−3^), 0.1728(g∙m^−3^), and 1.74. This wavelength was chosen as it showed good performance in the original study (coefficient of multiple determination, R^2^ = 78.9%) and also allows for better comparison with the second SPM calculation method, which is also based on values at 665 nm (although not exclusively).

The second SPM calculation method, proposed by Balasubramanian et al. [42], utilizes a hybrid approach, in which the water type is first identified, and then different processing steps are carried out for each type. The SOLID model starts with a semi-analytical step, in which the particulate backscattering (b_bp_) at 665 nm is estimated using the so-called quasi-analytical algorithm (QAA) [43]. SPM is then retrieved by an empirically calibrated power-law function, given by Equation (13):

(13) $$\mathrm{SPM}=53.736\times {\;b}_{\mathrm{bp}}{\left(665\right)}^{0.8559}$$

## 3. Results

### 3.1. Radiometric Data

**Figure 3:**
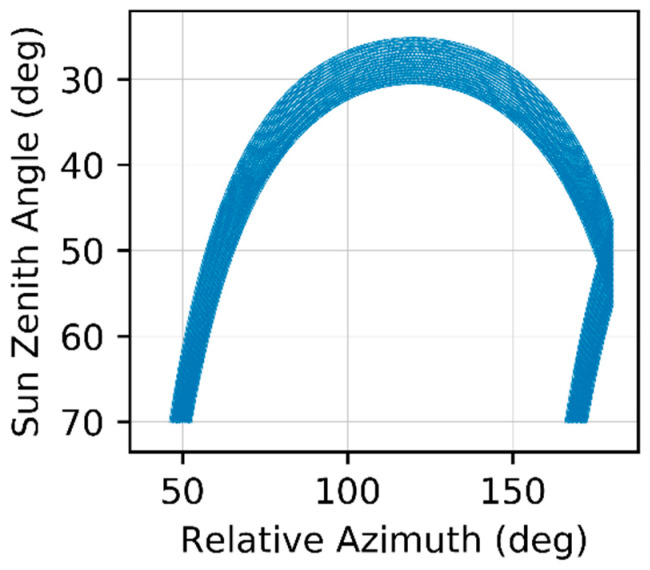
Sun zenith angle and sun-sensor relative azimuth corresponding to all radiometric measurements collected during the 18-day experiment.

Radiometric data were successfully acquired during the entire measurement period without interruption. Due to the presence of shadowing effects in the data collected during early morning and late afternoon, we only processed measurements in which the sun zenith angle was <70°, which resulted in a total of 18,371 measurement sets of E_d_, L_u_, and L_d_ in situ measurements. Figure 3 shows the variation of the sun zenith angle and sun-sensor relative azimuth during the measurement period, over which we observed a minimum sun zenith angle of ~25°.

**Figure 4:**
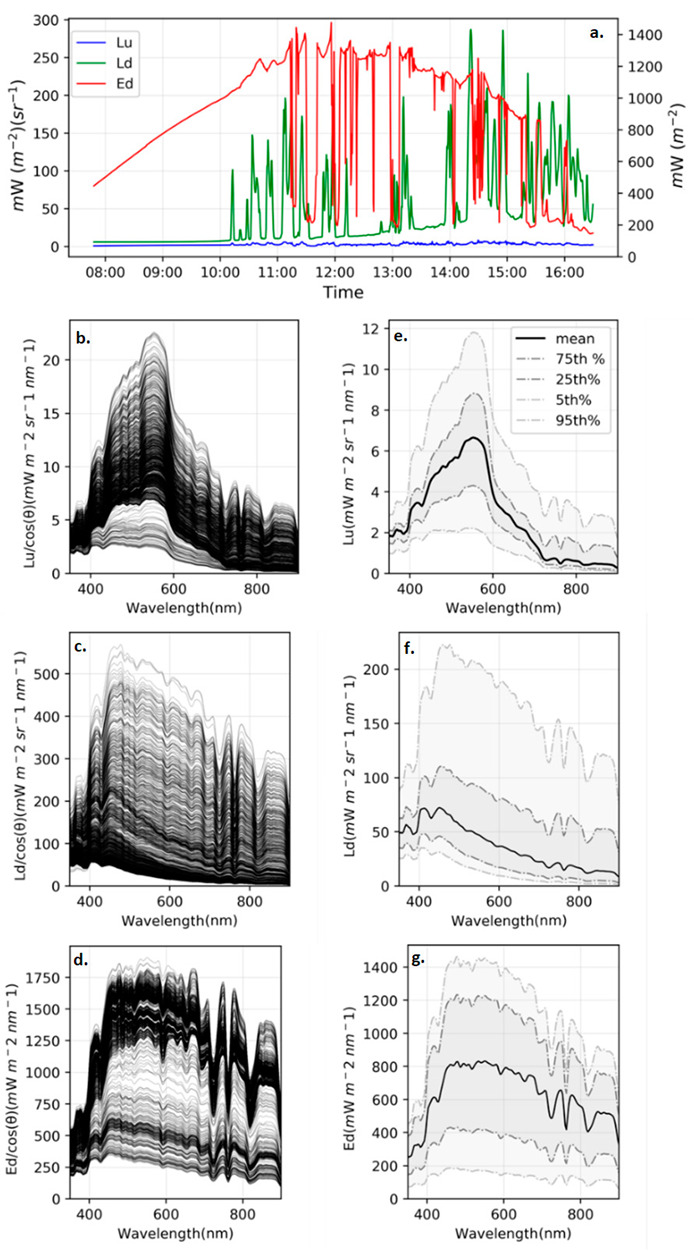
Radiometric measurements. Panel (**a**) shows radiometric values at 550 nm that were recorded on the 7th day of measurement, L_u_ and L_d_ values are shown on the main axis, and E_d_ values are on the secondary axis. Panels (**b**–**d**) show cosine-normalized radiometric spectra recorded on the 7th day of measurement. Panels (**e**–**g**) show percentile values of radiometric spectra for the whole dataset (all 18 days).

As data were collected throughout most of the day, there was considerable variability in the L_u_, L_d_, and E_d_ data (Figure 4). Most of the variability in the raw data was simply due to varying direct solar irradiance; however, significant variability was also induced by changing sky conditions (i.e., cloud coverage). Table 1, in which normalized data show a high coefficient of variance values for three different wavelengths, also shows this behavior. Figure 4a shows the L_u_, L_d_, E_d_, and R_rs_ time series at 550 nm from a whole day of acquisition. Morning sky conditions match clear-sky conditions until 10 a.m., showing steadily increasing E_d_ values and stable records for the other variables. From 10 a.m. to 3:30 p.m., we note strongly varying values of E_d_ (550) (varying between m246 Wm^−2^nm^−1^ and 1654 mWm^−2^nm^−1^) and L_d_ as a function of cloud coverage. For the latest time period until 4:30 p.m., we note a very low E_d_ (of about 200 W·m^−2^) but rather elevated L_d_ values. This shows how challenging it is to apply the correction of R_rs_ as a function of varying illumination geometry and/or cloud coverage.

**Figure 5:**
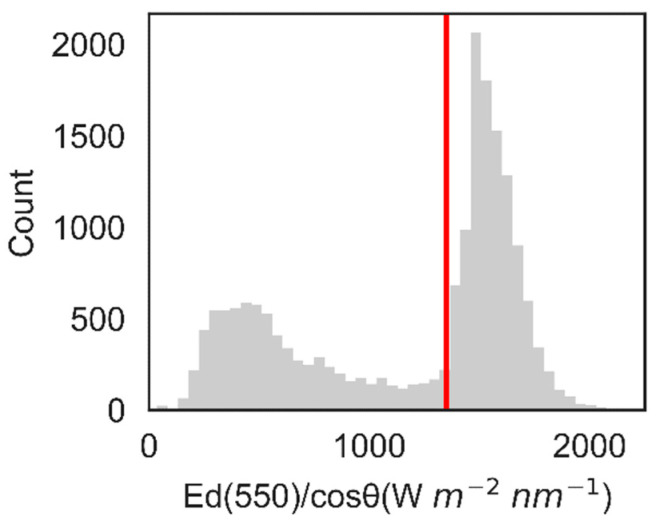
Histogram of cosine-normalized irradiance at 55 nm for the 18-day experiment. The red vertical line at 1350 mWm^−2^nm^−1^ shows the threshold used to distinguish clear-sky and cloudy classes.

A more detailed inspection of the intraday variability shows that there are periods of time in which the radiometric data stay stable and periods when they vary quite a lot. These variations, which occur even on successive data points (30 s intervals), coincide with variations in sky conditions, mostly related to cloud coverage. To more efficiently assess the gain of the different processing/correction schemes that were evaluated, we classified the dataset into three different categories. The first two categories are related to the measured and cosine-normalized E_d_ (which serves as a simple proxy for the expected clear-sky E_d_). This classification was determined based on the distribution of the cosine-normalized values of irradiance at 55 nm, as shown in Figure 5. The bimodal distribution was divided into two classes—clear sky and cloudy—using a chosen threshold of 1350 mWm^−2^nm^−1^.

We also classify measurement points in an additional class referred to as “ideal conditions”, which is a subset of the clear-sky class, corresponding to when there are optimal measurement conditions. In this class, data with a relative azimuth > 90° and sun zenith angle <50 ° were selected and then filtered to remove measurements with too many scattered clouds. To do this, measurements in which L_d_ values were higher than expected due to pointing at a cloud (L_d_(550) > 0.15 mWm^−2^nm^−1^sr^−1^) were removed, as was the top half of the clear-sky dataset (E_d_/cos θ > 1618 mWm^−2^nm^−1^). After classification, the clear-sky, cloudy, and ideal classes were composed of 10,771, 7600, and 2612 measurements, respectively.

### 3.2. Obtaining and Processing Remote-Sensing Reflectance

**Figure 6:**
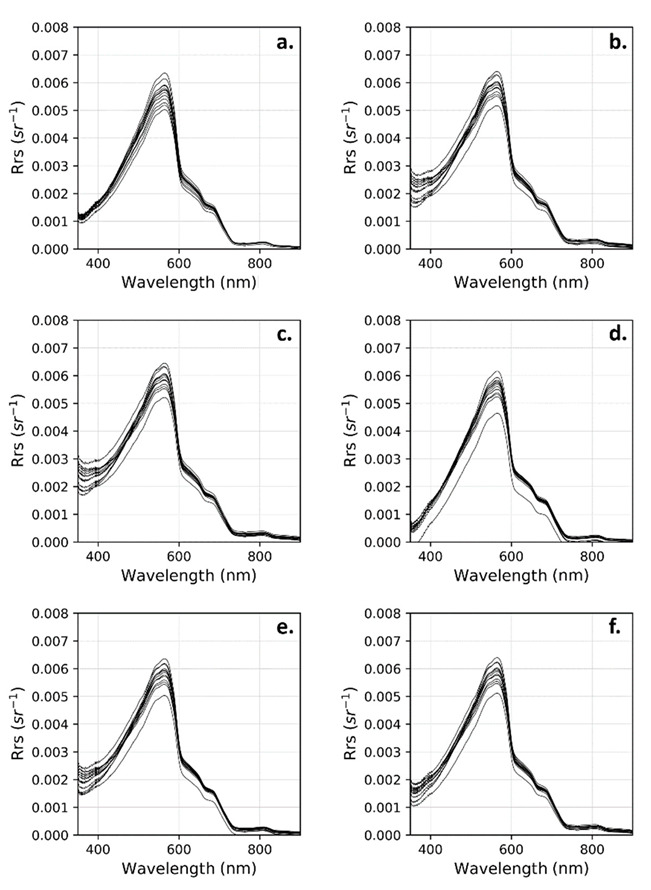
Reference R_rs_ spectra obtained by each calculation method used. (**a**) 3C; (**b**) M99(1); (**c**) M99(2); (**d**) M15; (**e**) OSOAA(fine); (**f**) OSOAA(coarse). Each curve is the reference R_rs_ of a day of measurement that corresponds to the best measurement acquisition conditions (relative azimuth > 90°, sun zenith angle < 50°, E_d_/cos θ > 1618 mWm^−2^nm^−1^, L_d_(550) < 0.15 mWm^−2^nm^−1^sr^−1^).

We defined a reference R_rs_ spectra for each day in order to validate the different R_rs_ calculation models. These reference spectra were selected using data obtained for each day during the best cloud-free conditions. As shown in Section 2.4, model validation was performed using data collected during ideal conditions from each measurement day. As such, Figure 6 shows all reference spectra used for the UPD calculation. As it can be seen, during the 18 measurement days, spectra collected in ideal conditions remained relatively stable. For example, the coefficient of variation of the reference R_rs_ for the M99 model was 7.4% at 44 nm, 4.6% at 55 nm, 5.0% at 66 nm, and 8.9% at 800 nm.

**Figure 7:**
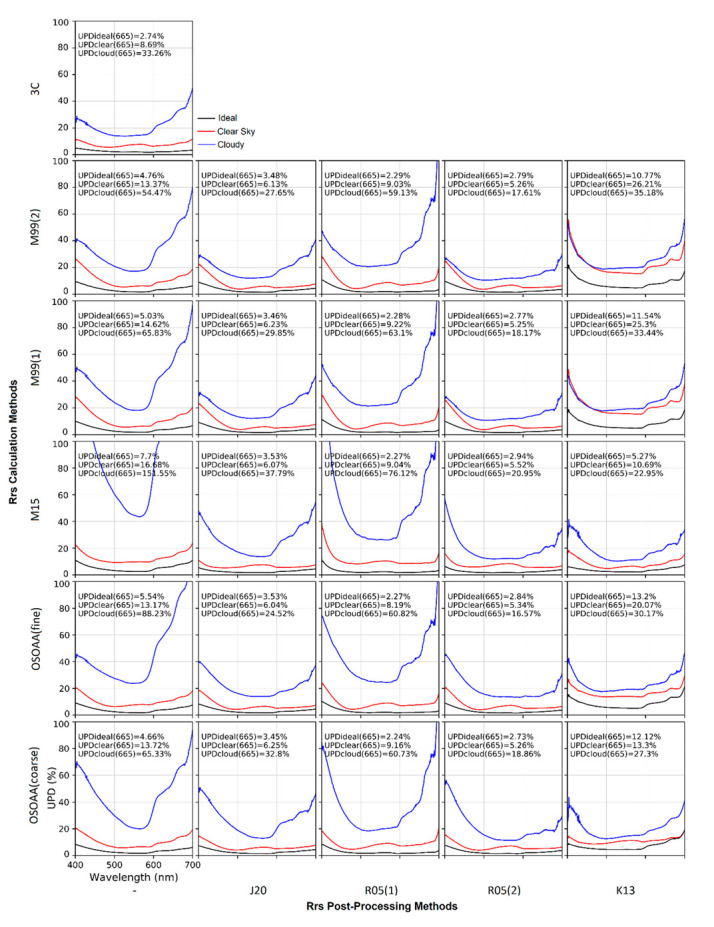
Each column corresponds to an R_rs_ postprocessing method, whereas each row corresponds to an R_rs_ calculation method. As such, each panel shows UPD(%) values of wavelengths in the 400–700 range for different model combinations and for different sky conditions (clear, cloudy, and ideal).

R_rs_ data were calculated and postprocessed using the different models described in Section 2.3.1 and Section 2.3.2. Each R_rs_ calculation and postprocessing scheme combination had its performance assessed, as described in Section 2.4. The results for ideal, clear-sky, and overcast conditions are shown in Figure 7 for the range between 400 nm and 700 nm. As expected, UPD values were the lowest in ideal and clear-sky conditions, and considerably higher in overcast conditions. For almost all methods, UPD values were lower in the 500 to 600 nm range, and higher when closer to 400 nm or 700 nm.

Amongst the R_rs_ calculation methods (without further processing, first column in Figure 7), 3C had the lowest overall UPD values in all situations analyzed. At 665 nm, we found that the UPD was 33.26%, 8.69%, and 2.52% for overcast, clear-sky, and ideal conditions, respectively, for this method. For ideal and clear conditions, the UPD was mostly stable throughout the analyzed spectral range; however, for overcast conditions, the UPD was considerably higher when closer to 400 nm or 700 nm.

The M99(1), M99(2), OSOAA(fine), and OSOAA(coarse) methods showed very similar UPD values in all conditions, especially in ideal and clear-sky conditions, in which differences between them were negligible. The UPD(665 nm) for overcast, clear-sky, and ideal conditions stayed within the ranges of 4.24–4.89%, 13.37–14.62%, and 54.47–88.23% for these four methods, respectively. Conversely, the M15 method had a markedly inferior performance in all conditions, although the UPD values for the clear-sky and ideal conditions were still satisfactory (i.e., lower than 17% at 665 nm).

A correction processing step was then applied to the R_rs_ obtained by each calculation method (except spectra obtained by the 3C method, as it already includes correction procedures), as shown in columns 2–6 of Figure 7. Both the R05(2) and the J20 algorithms showed significant reductions in UPD values when applied to calculation methods. Overall, R05(2) was slightly more successful. As an example, when it was applied to the M99(1) calculation method, The UPD(665 nm) for each weather condition was reduced from 65.83%, 14.62%, and 4.55% to 18.17%, 5.25%, and 2.54%, respectively. When applied to the same calculation method, J20 achieved UPD(665 nm) values of 29.85%, 6.23%, and 3.1%. In contrast, the R05(1) method only slightly improved UPD values, and even worsened them in some pairings. The K13 algorithm also had unsatisfactory results; even though it successfully reduced UPD values for overcast conditions, it significantly worsened the UPD for clear-sky and ideal conditions, i.e., when applied to OSOAA(fine), the UPD(665 nm) at ideal conditions went from 5.54% to 13.2%, except when it was applied to the M15 method (which curiously was the worst performing method on its own).

When looking at UPD(665 nm), only a small difference was observed between the different calculation methods when applied to the best-performing postprocessing algorithms (R05(2) and J20). However, when looking at lower wavelengths, both OSOAA methods had better results in ideal and clear-sky conditions while having worse results when overcast. When postprocessed by R05(2), the M99(2) method found the UPD(450 nm) of 15.0%, 9.7%, and 3.9% for overcast, clear-sky, and ideal conditions, respectively; when applying the same method, OSOAA(fine) found respective values of 26.6%, 3.6%, and 7.8%.

**Figure 8:**
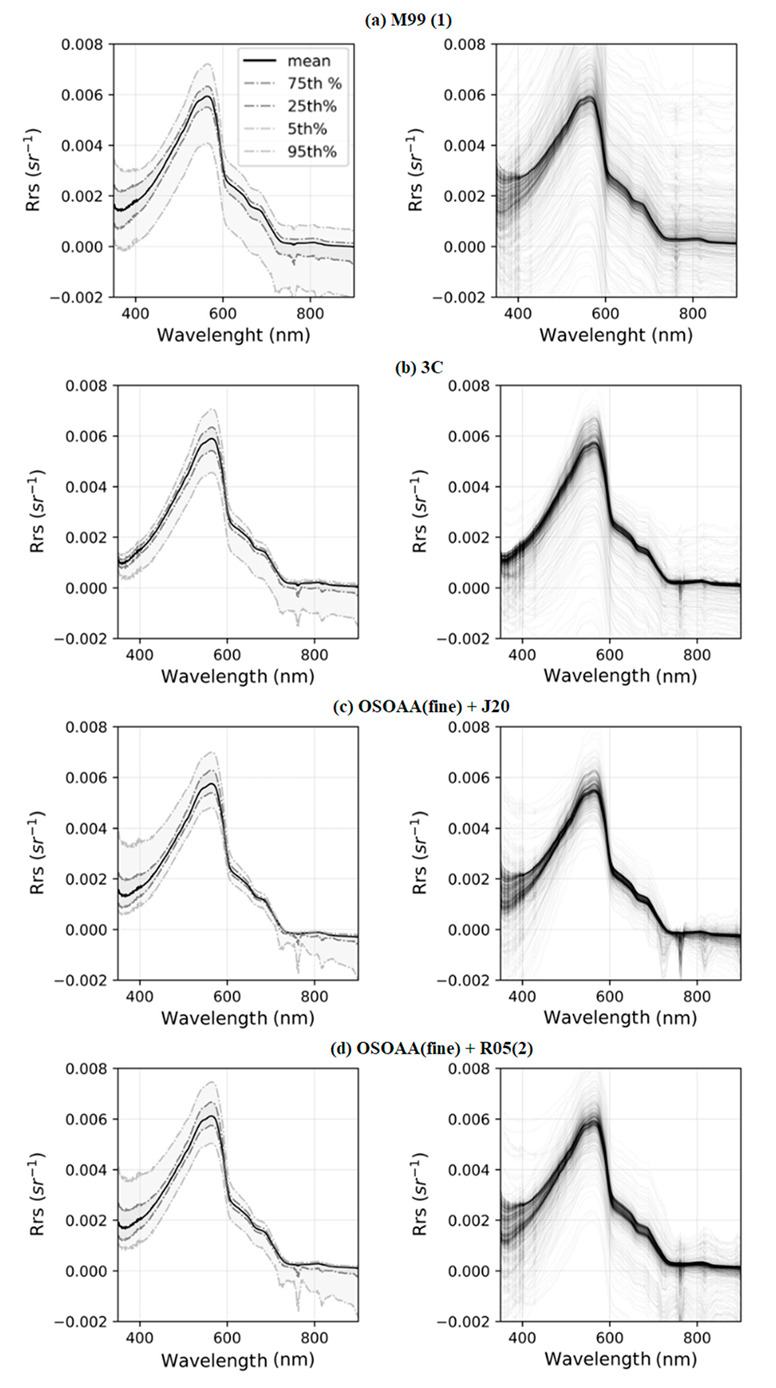
The first column shows spectra R_rs_ percentile for the whole dataset, and the second column shows all R_rs_ spectra obtained from data of the 7th day of measurement.

Percentile values (5%, 25%, 75%, and 95%) of the resulting spectra that had the lowest overall UPD values, as well as all spectra from day 7, are shown in Figure 8. In it, we can see that: (1) 3C achieved a low variance for spectra in the blue range, even in very varied weather conditions; (2) OSOAA(fine) + J20 and OSOAA(fine) + R05(2) have very similar resulting spectra percentiles; (3) in the >600 nm range, algorithms were very effective at correcting values above the median spectral values, with most of the observed variance being observed for values with a negative bias; and (4) the most significant variation was still observed closer to the reflectance peak at 550 nm.

### 3.3. Time-Series Smoothing

**Figure 9:**
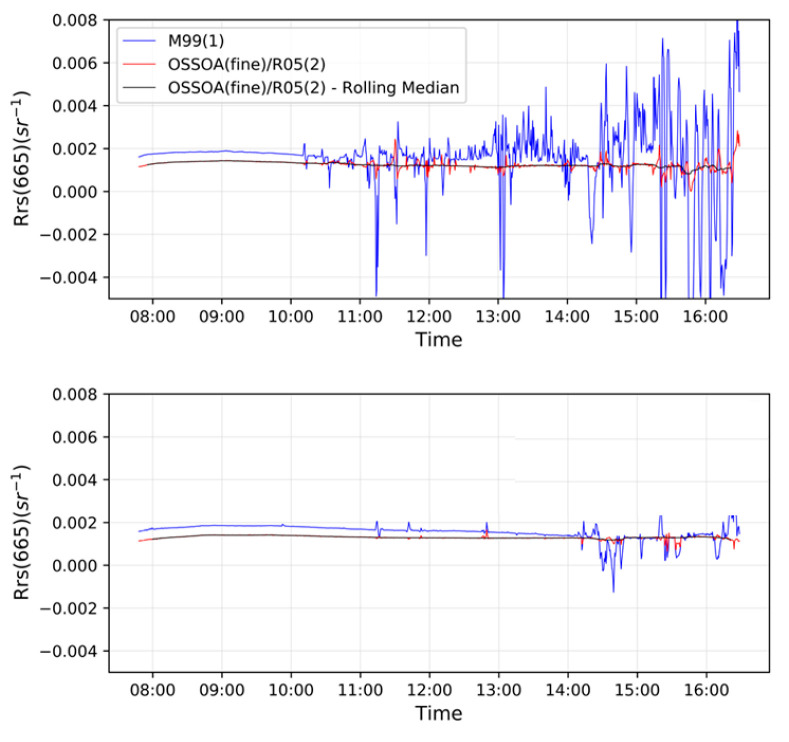
Comparison of R_rs_ at 665 nm, obtained using (i) M99(1) method; (ii) OSOAA(fine) postprocessed with R05 method; (iii) OSOAA(fine) postprocessed with R05 method with a final application of a 15 min rolling median filter. Data presented for the 7th (**above**) and 8th (**below**) day of measurement.

After obtaining processed spectra, we applied a simple 15 min window moving median to the time-series spectral data as a smoothing filter. Figure 9 shows the resulting time series for R_rs_(665 nm) for days 7 and 8 that compares the spectra obtained by M99(1), as well as that of OSOAA(fine)/R05(2) before and after smoothing. R_rs_ obtained by the M99(1) method, which is shown as a baseline reference, resulted in highly variable R_rs_ during cloudy conditions, which occurred during most of day 7. During day 7, R_rs_-M99(1) remained mostly stable until around 14:00, when some variation was observed due to the presence of scattered clouds. On the other hand, R_rs_ obtained with OSOAA(fine) and R05(2) shows some variability during cloudy conditions, but significantly less than what is observed when applying M99(1). After smoothing, variability was further reduced, with the resulting R_rs_(665) staying quite stable throughout the day. As such, UPD(665) values of the OSOAA(fine)/R05(2) model were reduced from 18.2% for overcast conditions to 10.4%, and from 5.3% to 4.6% and from 2.8% to 2.6% for clear-sky and ideal conditions, respectively, when applying the smoothing filter.

### 3.4. Variation Due to Sun Angular Position

**Figure 10:**
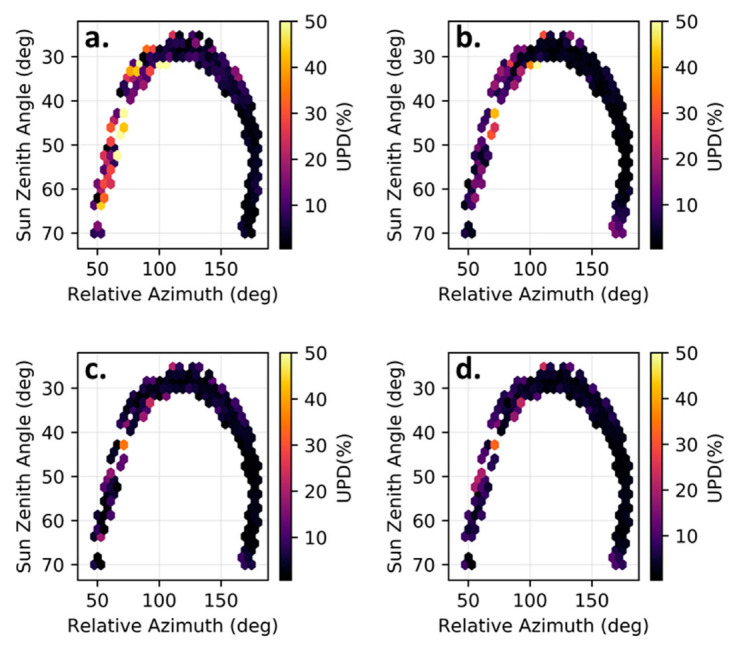
UPD(%) of different R_rs_ models measured in clear-sky conditions at different relative azimuth and sun zenith angles. (**a**) M99(1); (**b**) 3C; (**c**) OSOAA(fine) + J20; (**d**) OSOAA(fine) + R05(2).

In order to verify the model performance with regard to the variation of the incident solar angle throughout the day, Figure 10 shows mean UPD(%) values for four different processing/postprocessing combinations during clear-sky conditions. We found that, after processing, the UPD was also lowered in these conditions, especially due to an improvement during times when the relative azimuth was <90°. It can be readily seen that the M99(1) method performed poorly when the relative azimuth was lower than 90 degrees. However, the other three graphs show that the methods used were effective at improving data quality for low values of the relative azimuth and average UPD(665 nm) values for sun-sensor relative azimuths lower than 90°, which in clear-sky conditions were 36.9%, 20.5%, 14.3%, and 10.9% for the M99(1), 3C, OSOAA(fine) + J20, and OSOAA(fine) + R05(2) model combinations, respectively.

This suggests that, even though most R_rs_ measurement protocols recommend values of a relative azimuth close to 135°, or at the very least, higher than 90° [29,30], the usage of correction algorithms such as the ones applied in this study can extend the angular relative azimuth range in which spectral data can be collected using field radiometry. With regard to the effect of varying the sun zenith angle (which was only collected when lower than 70°), a significant degradation in spectral quality was not observed in the studied range, with only a very slight difference being observed for values higher than 65°. This performance for varying sun angles significantly increases the viability of a permanent radiometric station such as the one used in this study for the measurement of R_rs_ in inland water bodies.

### 3.5. SPM Estimation

Using spectra obtained by different methods, SPM was then estimated using the N10 and SOLID models. Table 2 shows the values of the mean SPM during the entire measurement period, as well as the coefficient of variation, for four different methods with and without the smoothing filter. In accordance with the findings regarding the different correction algorithms, the best-performing calculation/correction methods reduced the observed CV when compared to the baseline M99(1) model, while not changing the mean SPM significantly. Similarly, applying the smoothing filter also decreased the observed CV. More interestingly though, we found a significant disparity in the observed CV between the different SPM inversion methods used. Although mean values were similar (~2.3 g/m^3^ for N10 and ~1.7 for SOLID), the QAA-based SOLID algorithm had much lower values of CV. In fact, when combining all the best-performing processing steps (OSOAA(fine), R05(2), with the smoothing filter, applied to SOLID), we find a CV of only 2.7%, in contrast to a CV of 69.5% found when applying the simplest processing pipeline (M99(1) applied to N10). SPM was also determined from water samples collected during the experiment, in which the three water samples found an equal SPM concentration value of 1.0 g/m^3^.

## 4. Discussion

After all processing steps, the obtained spectra had their quality improved when compared to spectra obtained using the most straightforward processing methodology (M99(1)). In particular, the 3C method was highly effective in reducing the observed UPD, and although the other calculation methods did slightly improve spectra quality (except for M15 which worsened spectral errors), the best performance was found by applying the obtained spectra to correction algorithms. We found that both J20 and the R05(2) were sufficiently effective at reducing errors in all starting reflectance calculation methods. R05(1) did not significantly improve spectra quality, and K13 was only effective at correcting data collected during cloudy conditions. After applying correction algorithms, we found that the spectra resulting from both OSOAA methods had the overall best results with respect to the UPD, with satisfactory results in the whole spectrum range. When looking specifically at blue wavelengths, the 3C method also showed satisfactory results, especially in ideal and clear conditions, in which it was the best-performing method. This is consistent with what was reported by Groetsch et al. [39], who found that using a spectrally dependent correction offset was the most appropriate. In contrast, Jiang et al. [34] argued that the wavelength dependance of a correction offset is negligible. In a sense, our results support both affirmations, in that although there were considerable differences between models that used these differing approaches, these results were only significant at shorter wavelengths between 350 nm and 450 nm.

The best results were found when applying R05(2) to OSOAA(fine) spectra. Even before applying the smoothing filter, the resulting UPD is already satisfactory enough to be used as the valid R_rs_ for the estimation of water OACs. Using M99(1) as a comparison baseline, we can see how effective the error correction was, particularly during cloudy conditions, in which the UPD(665 nm) went from 88.23% to 18.17%. Although R05(2) showed great results in this study, the authors of the original study say that the model is limited to waters with relatively low concentrations of SPM, which is the regime in which the water similarity spectra can be applied. In contrast, both 3C and J20 were reportedly calibrated for a much larger SPM concentration range. Therefore, although R05(2) had better results in this study, different water conditions might yield better results for the other methods mentioned above.

The final processing step of applying a smoothing filter to the time-series data was also found to be highly effective at reducing observed errors. This points to a conclusion where a high proportion of observed variance is due to stochastic effects in measured data, which is mostly due to variation in cloud position and illumination and, to some extent, to radiometer measurement artefacts. However, the usage of such a filter does limit the information that can be collected from small time intervals, and thus, an appropriate filter window should be chosen to reflect the objectives of the measurement. In this study, we chose a 15 min window, as we found that it was sufficient to significantly decrease errors while still being much smaller than the interval in which water components typically change to a significant degree. Thus, different water body dynamics might justify a different postprocessing choice.

We also found that different state-of-the-art SPM inversion models can find significantly different results with regard to the variability of the observed data. When comparing the mostly empirical N10 algorithm to the SOLID algorithm, which is a single-blended algorithm that applies an empirical and analytical approach, we found that the SPM estimated using SOLID had much lower values of CV. Indeed, this reduction in the overall SPM variability found is probably because the SOLID model estimates SPM by applying an empirical expression to b_bp_ retrieved by QAA. Additionally, by being a physically based model, QAA can estimate b_bp_ independently of certain spectral variations, reducing the overall variability. However, even though these methods performed differently regarding observed variability, mean values were similar and in line with what was found with laboratory-analyzed water samples.

With regard to the SPM estimation, the relationship between OACs, water optical properties, and SPM can vary significantly depending on the specific properties of each individual water body, and for this reason, estimates derived from remote-sensing data will not be as accurate as other more direct measurements [44]. As such, the lack of a more detailed analysis of the accuracy of retrieved SPM concentrations is a limitation of this study as the SPM concentration results are based on the assumption that the existing SPM inversion algorithms can be applied to the studied environment. However, still, these estimates can provide valuable information to understand suspended sediment dynamics, and R_rs_-derived SPM data has been used in a variety of aquatic environments (e.g., coastal lagoons and estuarine areas [45], tropical rivers [46], river floodplains [47], river confluences [48], etc.).

Another important consideration regarding the findings of this study is that the model assessments did not use, as reference values, field-validated spectra that could be derived, for example, from subsurface reflectances. Instead, we used as a reference the R_rs_ spectra that were collected in ideal conditions during each measurement day. For this reason, the resulting UPD values for each model are best interpreted not necessarily with regard to finding the actually correct spectra, but more specifically for stabilizing the effects of changing illumination conditions over time. Additionally, water conditions, as well as R_rs_ spectra, measured during ideal conditions remained very stable throughout the duration of the study. Additionally, although this is very advantageous with regard to comparing calculation and correction algorithms, as the illumination variables could be better isolated, it also means that only a specific water condition was evaluated.

In summary, we found that there is a high potential for the application of autonomous radiometric measurement stations to measure optically active components in water. As such, a radiometric station could be used as a viable alternative in the monitoring of water bodies, such as reservoirs and rivers, and the high-frequency nature of the measured data could create further opportunities for the understanding of temporal dynamics of SPM, as well as other OACs. Although varying illumination conditions create challenges in the processing of spectral data, the combination of existing calculation, correction, and smoothing algorithms can considerably reduce errors in measured data. However, further studies are still necessary, particularly to understand how such a processing pipeline would fare in other water conditions.

## Figures and Tables

**Table 1:** Coefficient of variation for cosine-normalized radiometric measurements at selected wavelengths for the 18-day experiment.

	CV at 400 nm	CV at 550 nm	CV at 665 nm
L_u_/cos(θ)	29.7%	37.6%	60.2%
L_d_/cos(θ)	48.2%	86.2%	108.7%
E_d_/cos(θ)	41.2%	45.5%	48.0%

**Table 2:** Coefficient of variation and mean of SPM values obtained using different R_rs_ spectra and SPM inversion models.

		No Smoothing		With Smoothing	
R_rs_ Model	SPM Model	Mean (g/m^3^)	CV (%)	Mean (g/m^3^)	CV (%)
M99(1)	N10	1.74	69.5%	1.68	53.6%
	SOLID	2.23	17.5%	2.24	10.3%
3C	N10	1.76	43.8%	1.72	25.6%
	SOLID	2.24	10.7%	2.26	4.9%
OSOAA(fine) J20	N10	1.60	52.5%	1.58	30.4%
	SOLID	2.32	7.3%	2.33	3.9%
OSOAA(fine) R05(2)	N10	1.65	48.5%	1.63	29.4%
	SOLID	2.20	4.1%	2.2	2.7%

## Data Availability

Not applicable.
